# Editorial: Community series: systemic vasculitis: advances in pathogenesis and therapies, volume II

**DOI:** 10.3389/fimmu.2025.1766442

**Published:** 2026-01-06

**Authors:** Alexandre Wagner Silva De Souza, Joshua Daniel Ooi, Jun Deng, Stefania Croci, Poh-Yi Gan, Kelly L Brown

**Affiliations:** 1 Rheumatology Division, Department of Medicine, Universidade Federal de São Paulo – Escola Paulista de Medicina, São Paulo, SP, Brazil; 2 Centre for Inflammatory Diseases, Department of Medicine, Monash Medical Centre, School of Clinical Sciences, Monash University, Clayton, VIC, Australia; 3 Henji Hospital, School of Medicine, Shanghai Jiaotong University, Shanghai, China; 4 Unit of Clinical Immunology, Allergy and Advanced Biotechnologies, Azienda Unità Sanitaria Locale-Istituto di Ricovero e Cura a Carattere Scientifico (IRCCS), Reggio Emilia, Italy; 5 Centre for Inflammatory Diseases, Monash University, Clayton, VIC, Australia; 6 BC Children’s Hospital Research Institute, Centre for Blood Research, and Department of Pediatrics, University of British Columbia, Vancouver, BC, Canada

**Keywords:** adaptive immunity, innate immunity, pathophysiology, systemic vasculitis, vaccination

Systemic vasculitis is a group of rare and multisystem diseases characterized by inflammation of blood vessels of different types and sizes (1). The etiology of systemic vasculitis is poorly understood. However, different immunological mechanisms have been recognized to play a role in its pathogenesis. These mechanisms include granuloma formation, neutrophilic or eosinophilic inflammation, immune complex deposition on vessel walls, and the presence of pathogenic autoantibodies, such as antineutrophil cytoplasmic antibodies (ANCA) and anti-glomerular basement membrane (anti-GBM) antibodies (2–8).

Vasculitis is classified according to the size of the blood vessels predominantly affected by the inflammatory process as large-, medium-, small-, and variable-vessel vasculitis. Vasculitis may affect a single organ or multiple organ systems simultaneously. It may also develop secondary to other systemic diseases such as systemic lupus erythematosus or rheumatoid arthritis. Infectious diseases, malignancies, and drug use, including illicit drug abuse, are also recognized as causes of vasculitis (9). People living with vasculitis experience protean manifestations due to the involvement of different organs and systems in various combinations (1).

In this Research Topic, Biegelmeyer et al. conducted a multicenter prospective cohort study to evaluate the response to primary and booster doses of the SARS-CoV-2 vaccination in patients with systemic vasculitis. They included 73 patients, 60 of whom completed the booster dose. Most of the patients had Behçet’s disease (BD), Takayasu arteritis (TAK), and antineutrophil cytoplasmic antibody (ANCA)-associated vasculitis (AAV). After the primary vaccination schedule of two doses, the ChAdOx1 nCoV-19 vaccine induced higher levels of IgG anti-RBD (SARS-CoV-2 spike receptor-binding domain) than the CoronaVac vaccine. However, no differences in immunogenicity were observed after the booster dose. Additionally, the immunogenicity of heterologous and homologous vaccination regimens or vasculitis forms was similar. Following vaccination, rates of new cases of COVID-19, hospitalization, and mortality were relatively low. There was no increase in the relapse rate, and adverse events were mostly mild. In TAK patients, Zarur et al. evaluated whether single-nucleotide polymorphisms (SNPs) of genes involved in homocysteine metabolism were responsible for the observed hyperhomocisteinemia (10). Using Sanger sequencing, they tested the following SNPs: C677T (rs1801133) and A1298C (rs1801131) in the *MTHFR* gene, A2756G (rs1805087) in the *MTR* gene, A66G (rs1801394) in the *MTRR* gene, and G80A (rs1051266) in the *SLC19A1* gene. They evaluated these SNPs in 73 TAK patients and 71 controls. Despite TAK patients having higher mean homocysteine levels than controls, the frequency of the SNPs carriage was similar in both groups. Takayasu arteritis itself was an independent risk factor for hyperhomocysteinemia, and thiazide diuretic use was an additional risk factor for increased homocysteine levels among TAK patients. Neither homocysteine levels nor SNP carriage was associated with ischemic events. The authors hypothesized that the inflammatory burden of TAK may be a risk factor for the hyperhomocysteinemia observed in this disease.

In AAV patients, Wu et al. conducted a case series of reduced-dose obinutuzumab in 16 adult patients with AAV who were refractory to cyclophosphamide or rituximab induction therapy or were treatment-naïve with multiorgan failure. The complete response rate at week 76 was 81.3%, and only one patient relapsed during follow-up. Despite the high frequency of treatment-emergent infections (43.8%), no severe infections occurred. Obinutuzumab is a promising type II anti-CD20 that is currently being tested in AAV patients, and these preliminary data highlight its potential effectiveness.

Furthermore, three review articles were published on this Research Topic, each approaching different aspects of the pathophysiology and management of vasculitis, particularly AAV (Zhang et al., Alberici et al., Tay et al.). Zhang et al. discuss the role of anti-endothelial cell antibodies (AECAs) in the pathogenesis of various forms of vasculitis. In their review, they describe the antigen targets of AECAs and the pro-inflammatory, pro-coagulant, and pro-apoptotic effects that AECAs trigger upon binding to endothelial cells. They also discuss how these effects contribute to the pathogenesis of systemic vasculitis. Alberici et al. reviewed the management of AAV patients, highlighting the burden of disease relapses and their risk factors, including clinical and exploratory biomarkers. They also reviewed AAV relapses and the treatment options for preventing and treating relapses in detail. Tay et al. reviewed the pathogenesis of myeloperoxidase (MPO)-AAV and the current therapeutic options for AAV. They also discussed emerging cell-based therapies, including the promising regulatory T (Treg) cell therapy, which involves the genetic engineering of Treg cells. These therapeutic modalities include TCR-Treg cells and CAR-Treg cells.

In addition to the original studies and review articles, three manuscripts detailing unusual case reports were published under this Research Topic. Rivet et al. presented a case-based literature review of three patients who developed severe digital necrosis while undergoing Immune Checkpoint Inhibitor (ICI) therapy with pembrolizumab, nivolumab, or a combination of nivolumab and ipilimumab for lung adenocarcinoma, renal cell carcinoma, or melanoma, respectively. The authors also identified 12 additional cases reported in the literature and described the primary characteristics of this severe ICI-related complication in all 15 cases. Zhang et al. reported rare and severe gastrointestinal manifestations of IgA vasculitis (IgAV) such as pancreatitis and esophageal necrosis in a 35-year-old male patient. The patient died due to severe gastrointestinal bleeding, hemorrhagic shock, and disseminated intravascular coagulation. Finally, Zhang et al. described a patient with long-standing Behçet’s disease due to trisomy 8 and myelodysplastic syndrome. The patient developed an unusual combination of IgA nephropathy and acute tubular necrosis, which progressed to severe renal failure requiring renal replacement therapy. However, renal function recovered after receiving methylprednisolone therapy.

In conclusion, this Research Topic comprises original articles that investigate different topics related to systemic vasculitis, including the immunogenicity to SARS-CoV-2 vaccination in patients with vasculitis, the genetic basis for hyperhomocysteinemia in TAK, and the response to obinutuzumab therapy in AAV patients. Moreover, this Research Topic reviews important issues in systemic vasculitis, such as the pathogenesis by AECAs, the risk of relapse, and the management of AAV, as well as emerging cell-based therapies for AAV patients. We hope this Research Topic provides useful information for the clinical management of systemic vasculitis.
